# Crossbreeding East African Highland Bananas: Lessons Learnt Relevant to the Botany of the Crop After 21 Years of Genetic Enhancement

**DOI:** 10.3389/fpls.2019.00081

**Published:** 2019-02-05

**Authors:** Michael Batte, Rony Swennen, Brigitte Uwimana, Violet Akech, Allan Brown, Robooni Tumuhimbise, Helena Persson Hovmalm, Mulatu Geleta, Rodomiro Ortiz

**Affiliations:** ^1^International Institute of Tropical Agriculture, Kampala, Uganda; ^2^Department of Plant Breeding, Swedish University of Agricultural Sciences, Alnarp, Sweden; ^3^International Institute of Tropical Agriculture, The Nelson Mandela African Institution of Science and Technology, Arusha, Tanzania; ^4^Laboratory of Tropical Crop Improvement, Katholieke Universiteit Leuven, Leuven, Belgium; ^5^Bioversity International, Heverlee, Belgium; ^6^National Agricultural Research Organization, Kampala, Uganda

**Keywords:** East African highland bananas, embryo culture, genetic enhancement, *Musa acuminata*, NARITA cultivars, pollination success, secondary triploids, seed set

## Abstract

East African highland bananas (EAHB) were regarded as sterile. Their screening for female fertility with “Calcutta 4” as male parent revealed that 37 EAHB were fertile. This was the foundation for the establishment of the EAHB crossbreeding programs by the International Institute of Tropical Agriculture (IITA) and the National Agricultural Research Organization (NARO) in Uganda in the mid-1990s. The aim of this study was to assess the progress and efficiency of the EAHB breeding program at IITA, Sendusu in Uganda. Data on pollinations, seeds generated and germinated, plus hybrids selected between 1995 and 2015 were analyzed. Pollination success and seed germination percentages for different cross combinations were calculated. The month of pollination did not result in significantly different (*P* = 0.501) pollination success. *Musa acuminata* subsp. *malaccensis* accession 250 had the highest pollination success (66.8%), followed by the cultivar “Rose” (66.6%) among the diploid males. Twenty-five EAHB out of 41 studied for female fertility produced up to 305 seeds per pollinated bunch, and were therefore deemed fertile. The percentage of seed germination varied among crosses: 26% for 2*x* × 4*x*, 23% for 2*x* × 2*x*, 11% for 3*x* × 2*x*, and 7% for 4*x* × 2*x*. Twenty-seven NARITA hybrids (mostly secondary triploids ensuing from the 4*x* × 2*x*) were selected for further evaluation in the East African region. One so far –“NARITA 7”– was officially released to farmers in Uganda. Although pollination of EAHB can be conducted throughout the year, the seed set and germination is low. Thus, further research on pollination conditions and optimization of embryo culture protocols should be done to boost seed set and embryo germination, respectively. More research in floral biology and seed germination as well as other breeding strategies are required to increase the efficiency of the EAHB breeding program.

## Introduction

Banana (*Musa* spp.) is an important staple food and cash crop in the Great Lakes Region of East Africa (Pillay et al., 2001). Bananas originated in South East Asia and Indochina (Simmonds, 1962), where the earliest domestication of bananas occurred. According to Price (1995), bananas were introduced into East Africa by Arab traders over 2000 years ago. Perrier et al. (2018) stated, however, that East African highland bananas (EAHB) were rather introduced by Austronesian-speaking peoples who colonized the Indian Ocean islands, particularly Madagascar, and reached the East African coasts. Somatic mutations gave rise to the large variability in the EAHB, making East Africa the secondary center of diversity for this banana group. In Uganda, the EAHB dominate the production, accounting for 85% of the total area under banana production, while the exotic cultivars contribute with 15% (Tushemereirwe et al., 1996). The region produces more than a half of the total bananas produced in Africa. The EAHB are principally used for cooking and a small percentage for brewing. The cultivars used for cooking are commonly known as “Matooke” in Uganda while those used for brewing are called “Mbidde.” The cooking types are cooked/steamed when green, while the brewing types are brewed when ripe. The EAHB are the primary staple crop for over 13 million Ugandans (≈35% of the population), whose per capita consumption of 0.7 kg daily is the highest in the world. Given its perennial nature and year-round harvesting character, banana is an important food security crop (Nyine et al., 2017). The crop has increasingly become an important local cash crop. Its production declined over the past three decades due to pests like the banana weevil (*Cosmopolites sordidus)* and burrowing nematode *(Radopholus similis)*; pathogens, of which the most important are *Pseudocercospora fijiensis*, causing black Sigatoka or black leaf streak disease (Arango Isaza et al., 2016; Alakonya et al., 2018), *Fusarium oxysporum* f. sp. *cubense* causing fusarium wilt or Panama disease (Viljoen et al., 2017)*, Xanthomonas campestris* pv. *musacearum* producing banana bacterial wilt (Nakato et al., 2018), declining soil fertility and drought (Swennen et al., 2013). Breeding for host plant resistance was identified as a sustainable method for addressing the banana production challenges (Tushemereirwe et al., 2015). The International Institute of Tropical Agriculture (IITA) together with Uganda’s National Agricultural Research Organization (NARO) started a banana crossbreeding program in the mid-1990s. The present study provides insights into the first 21 years of crossbreeding EAHB and outlines progress made to overcome unique botanical challenges encountered in the breeding system of bananas.

The first efforts toward banana research in the East African region can be traced back to 1948, when a survey was conducted in Kenya, Uganda, Tanganyika and Zanzibar, under the auspices of the Banana Research Scheme (Baker and Simmonds, 1951). The aim of this survey was to find out if there were banana cultivars in East Africa of potential practical or scientific value for a breeding scheme, and to investigate the general botany of the wild bananas in the region. This survey started in Nairobi, then moved around Mount Kenya, to Kisumu, Entebbe, Fort Portal, Bukoba, Dar-es-Salaam, Zanzibar, Amani, Moshi and finally back to Nairobi. During the survey, local cultivars were grouped according to whether they were “native” or “alien” depending on the time the cultivars had been grown. Native cultivars were those that had stayed in communities for a long time, while the aliens were recently introduced. Entries were made by local names, followed by meaning of the name, usage, distinctive characters, and synonyms. From the survey, an inventory of banana diversity was developed comprising 247 accessions, 89 of which were EAHB according to the descriptions (Baker and Simmonds, 1952). Samples were sent to the Imperial College of Tropical Agriculture in Trinidad and used for research that generated knowledge about banana taxonomy and diversity, which is the basis of today’s banana breeding and genetic analysis.

Karamura (1998) investigated in-depth 238 accessions of the EAHB in the 1990s. The Ugandan collection of this germplasm faced a challenge of synonyms, since the same cultivar was called by different names depending on the region where it was grown. This prompted the development of a list of synonyms for EAHB in order to harmonize naming and eliminate duplicates. Consequently, 84 distinct clones were identified from the collection. The EAHB were grouped as “Mutika-Lujugira” (Baker and Simmonds, 1952; Shepherd, 1957), containing both cooking (known as “matooke” in Uganda) and beer/juice cultivars. The “Mutika-Lujugira” group was further divided into five clone sets based on their morphological characteristics, namely, Musakala, Nakitembe, Nakabululu, Nfuuka, and Mbidde (Karamura, 1998; Karamura and Pickersgill, 1999; Batte et al., 2018). The first four clone sets contain cooking cultivars while the fifth one contains beer/juice cultivars. The Musakala clone set comprises cultivars with lax bunches, while the Nakitembe clone set comprises cultivars with persistent neutral flowers on the rachis and Nakabululu clone set has cultivars with very compact bunches. The Mbidde clone set comprises cultivars that produce juice/beer while the Nfuuka clone set includes the remaining EAHB cultivars.

The next step toward breeding of EAHB was the screening of available germplasm for female fertility (Ssebuliba et al., 2005), despite the assumption that they are completely sterile. The highly pollen fertile diploid *Musa acuminata* subspecies *burmannica* known as “Calcutta 4” was used as male parent. Thirty-seven out of the 78 tested EAHB cultivars were found to set seed with a maximum of 25 seeds per pollinated bunch (Ssebuliba et al., 2005). Vuylsteke et al. (1993) and Talengera et al. (1996) indicated that hybrid production of cultivated triploid *Musa* clones was hampered by low seed germination rates. They pointed out that only 1% of hybrid plantain or banana hybrid-derived seed germinates when planted in the soil and that aseptic embryo culture increases rates of seed germination by a factor of 3 to 10%. Hence, EAHB seeds were germinated through embryo culture.

Breeding of triploid EAHB passed through an intermediary step whereby they were first crossed with diploids to obtain tetraploids, and later those tetraploids were further crossed with diploids to obtain secondary triploids (Brown et al., 2017). Diploid breeding clones were therefore introduced from the IITA breeding program in Nigeria and the Fundación Hondureña de Investigación Agrícola (FHIA, Honduras). After a series of crosses between the triploids, tetraploids and imported diploids, some diploid-derived breeding clones were generated, which were also incorporated in the crossing schemes. These new diploids were more desired because they showed EAHB (“matooke”) traits that could be inherited by the offspring, thereby increasing the chance of fruit acceptability by farmers of newly bred cultivars.

The aim of this study was to assess the progress and efficiency of the breeding program for the EAHB over the first 21 years, using the available data collected throughout the breeding pipeline at the IITA station in Uganda. This assessment of progress and challenges in the EAHB breeding program captures therefore past progress and provides insights to seek further improvements in the breeding for improved banana cultivars.

## Materials and Methods

In order to determine female fertility in EAHB, 41 cultivars belonging to five different clone sets were assessed (Table 1). Other 10 tetraploid hybrids derived from the EAHB program were also assessed for female fertility (Table 2). These were crossed with wild and imported diploid male parents from IITA and FHIA breeding programs and later with some diploids developed from hybridization between the EAHB and wild or improved diploids (Supplementary Table 1). The diploids used had superior breeding attributes like host plant resistance or tolerance to black sigatoka, *Fusarium* wilt, weevils and nematodes, parthenocarpic fruits, “Matooke” traits, yellow pulp, quick maturity, big bunch size, short stature, and drought tolerance (Supplementary Table 2). Further intercrossing between diploids and diploids, tetraploids and triploids were also pursued. The parental materials were planted in pollination blocks at Sendusu-Namulonge in Uganda (00°31′ 47″ N and 32°36′ 9″ E). Plant density was 1667 plants ha^−1^, with a spacing of 3 m between rows and 2 m among plants within a row.

**Table 1:** Female fertility and hybridization success in 41 East African highland banana cultivars crossed with different males from April 1995 to December 2015 at IITA-Sendusu, Uganda.

Clone set	Cultivar	Bunches pollinated (#)	Seeds (#)	Bunches without seed (#)	Highest seeds per bunch (#)	Mean of seeds per bunch ±*SE*	Pollination success (%)
Nakabululu	Bukumu	27	1	26	1	0.03 ± 0.03	3.7
	Kazirakwe	1043	599	891	30	0.6 ± 0.1	14.6
	Kibuzi	44	3	43	3	0.1 ± 0.1	2.3
	Mukubakkonde	18	0	18	0	0	0.0
	Nakabululu	35	54	23	10	1.5 ± 0.5	34.3
	Nakasabira	1567	1875	1245	305	1.2 ± 0.2	20.6
	Nakayonga	924	542	781	28	0.6 ± 0.1	15.5
	Nakyetengu	777	957	654	147	1.2 ± 0.2	15.8
	Salalugazi	18	0	18	0	0	0.0
	Total	4,453	4,031	3,699	305	0.9 ± 0.01	16.9
Nfuuka	Bitambi	183	21	174	6	0.1 ± 0.04	4.9
	Entukura	2264	1169	2011	47	0.5 ± 0.1	11.2
	Enyeru	1651	491	1524	36	0.3 ± 0.04	7.7
	Enzirabahima	2277	1537	2015	102	0.7 ± 0.1	11.5
	Kabucuragye	1405	300	1310	23	0.2 ± 0.03	6.8
	Kibalawo	30	16	24	4	0.5 ± 0.2	20.0
	Kulwoni	29	0	29	0	0	0.0
	Nabusa	661	115	634	21	0.2 ± 0.1	4.1
	Nakawere	38	53	26	13	1.4 ± 0.5	31.6
	Nakinyika	33	9	31	6	0.3 ± 0.2	6.1
	Namwezi	1117	401	1012	29	0.4 ± 0.1	9.4
	Nante	234	10	229	4	0.04 ± 0.02	2.1
	Nassaba	30	0	30	0	0	0
	Ndibwabalangira	41	20	36	8	0.5 ± 0.2	12.2
	Nfuuka	1088	339	995	23	0.3 ± 0.04	8.6
	Siira	31	0	31	0	0	0
	Tereza	2609	2107	2239	201	0.8 ± 0.1	14.2
	Total	13,721	6,588	12,350	201	0.5 ± 0.006	10.0
Nakitembe	Mbwazirume	29	0	29	0	0	0.0
	Nakitembe	479	16	473	9	0.03 ± 0.01	1.3
	Nandigobe	33	3	31	2	0.1 ± 0.1	6.1
	Nyamwihogora	14	5	12	3	0.4 ± 0.2	14.3
	Total	555	24	545	9	0.04 ± 0.008	1.8
Musakala	Mayovu	9	0	9	0	0	0
	Mukazialanda	21	0	21	0	0	0
	Murure	17	0	17	0	0	0
	Musakala	18	0	18	0	0	0
	Muvubo	41	0	41	0	0	0
	Nakibizzi	10	0	10	0	0	0
	Namunwe	22	1	21	1	0.04 ± 0.04	4.5
	Total	138	1	137	1	0.007 ± 0.007	0.7
Mbidde	Endirira	26	0	26	0	0	0
	Kabula	19	0	19	0	0	0
	Nalukila	27	0	27	0	0	0
	Nsowe	20	0	20	0	0	0
	Total	92	0	92	0	0	0

**Table 2:** Female fertility and hybridization success among 10 tetraploid bred hybrids crossed with different males from April 1995 to December 2015 at IITA-Sendusu, Uganda.

Tetraploid female	Bunches pollinated (#)	Seeds (#)	Bunches without seeds (#)	Highest seeds per bunch (#)	Mean seeds per bunch ±*SE*	Pollination success (%)
1201K-1	1656	48012	856	722	29.0 ± 1.9	48.4
917K-2	2373	93032	1230	2279	39.2 ± 2.5	48.2
660K-1	1746	25776	987	454	14.8 ± 1.0	43.5
222K-1	472	7818	279	450	16.6 ± 2.0	40.9
1438K-1	1591	30706	989	885	19.3 ± 1.6	37.8
365K-1	670	7687	455	284	11.5 ± 1.3	32.1
376K-7	736	7161	543	467	9.7 ± 1.4	26.2
401K-1	727	4811	535	348	6.6 ± 1.0	26.0
2180K-6	153	124	136	50	0.8 ± 0.4	11.1
199K-4	293	64	280	12	0.2 ± 0.1	4.4

Pollen from male parents were always collected around 07.00 h from flowers previously covered with cotton bags at anthesis. This was done to prevent pollen contamination from other pollen sources due to insects, bats, or birds (Mutsaers, 1993). Likewise, emerging inflorescences of female parents were bagged with transparent plastic bags, to avoid natural crossing with alien pollen until the last flower was pollinated. Hand pollinations were performed daily between 07.30 and 10.30 h on freshly exposed female flowers by rubbing a cluster of male flowers containing pollen onto the female flowers. Pollinated bunches were labeled with tags.

Bunches were harvested at maturity – when the first fruit started yellowing – and ripened in a store room until all fruits became yellow and the pulp was soft. Seeds were extracted subsequently and immediately sent to the tissue culture laboratory for embryo culture. This was done because the seeds produced by bananas and plantains are reported to have a high degree of dormancy such that direct germination in the soil is unfeasible (Ortiz and Vuylsteke, 1995). Embryos were excised and germinated *in vitro* according to Vuylsteke et al. (1990). *In vitro* seedlings were transferred to the greenhouse nursery for weaning, and ploidy level was determined for each genotype by flow cytometry. Aneuploids or hyperploids (pentaploids and above) were discarded because these hybrids exhibited gross abnormal foliage or stunted growth.

The following data were taken from April 1995 to December 2015 and used in the analyses: Pollination/crossing number, female parent, ploidy of the female parent, male parent, ploidy of male parent, date of pollination, harvest date, date of seed extraction, total number of seeds, number of good seeds (with black hard integuments), number of bad seeds (with brown soft integuments), date of embryo extraction, number of extracted embryos, number of germinated embryos after two months, contaminations, number of weaned plants, and number of hardened plants ready for planting in the field. The total number of seeds per cultivar, total number of bunches of a cultivar pollinated but without seed, highest seed per pollinated bunch, mean of seeds per bunch and standard errors, and pollination success for triploid and tetraploid cultivars were calculated. Likewise, the total number of seeds per diploid used as male to pollinate cultivars, total number of bunches of cultivars pollinated by a particular diploid male but without seed, highest seed per bunch when pollinated by a particular diploid male, mean of seeds per bunch pollinated by particular diploid male and standard errors, and pollination success for 29 diploid males were calculated. The pollination success throughout the 21 years under study was computed as:

$$\mathrm{Pollination success}=\frac{\mathrm{Number of pollinated bunches with seed}\times 100}{\mathrm{Total number of pollinated bunches}}$$

A one-way analysis of variance (ANOVA) at 95% confidence level was done to study the effect of month on pollination success using R-software version 3.4.1 (R Core Team, 2017). The mean number of seeds per cross type and standard errors, mean embryos extracted and standard errors, mean embryos germinated and standard errors were calculated, and seed embryo germination success per cross type over a period of 11 years (from 2005 to 2015) was obtained as:

$$\mathrm{Seed embryo germination success}=\frac{\mathrm{Number of germinated embryos}\times 100}{\mathrm{Total number of extracted embryos}}$$

## Results

### Pollination Success and Seed Set

Total annual pollination success was higher in some years than in others, the highest being in 2010 (37.23%) and lowest in 1995 (5.87%). Results from the ANOVA at 95% confidence level revealed, however, that the month of pollination did not result in significantly different (*P* = 0.501) pollination success over the 21-year period under study (Table 3). A great fluctuation in seed set among cultivars after crossing EAHB with wild or improved diploids, ranging from 0 to 305 seeds per pollinated bunch, was also observed. Generally, cultivars in the Nakabululu clone set had the highest pollination success (16.9%) followed by cultivars in the Nfuuka clone set (10%), then the Nakitembe clone set (1.8%) and the Musakala clone set (0.7%). The Mbidde clone set had zero pollination success (Table 1).

**Table 3:** Analysis of variance for pollination success (%) of each month for banana genotypes pollinated from April 1995 to December 2015 at IITA-Sendusu, Uganda.

Source of variation	DF	SS	MS	F_c_	*P* > F_c_
Months	11	1205	109.5	0.941	0.501 NS
Residuals	236	27458	116.3		

*DF, degrees of freedom; SS, sum of squares; MS, mean squares; F_c_, F calculated; NS, not significant.*

The highest pollination success for the EAHB cultivars was expressed by cultivar “Nakabululu” (34.3%) (Nakabululu clone set) with an average of 1.5 seeds per pollinated bunch, followed by “Nakawere” (31.6%) (Nfuuka clone set) with an average of 1.4 seeds per pollinated bunch, “Nakasabira” (20.6%) (Nakabululu clone set) with an average of 1.2 seeds per pollinated bunch, “Kibalawo” (20%) (Nfuuka clone set) with an average of 0.5 seeds per pollinated bunch and “Nakyetengu” (15.8%) (Nakabululu clone set) with an average of 1.2 seeds per pollinated bunch.

Tetraploid hybrids generated from EAHB and wild or improved diploids produced, on average, more seeds per pollinated bunch (Table 2) than their triploid parents (Table 1). The highest number of seeds per bunch was 2279, produced by “917K-2”. The highest pollination success for tetraploid females was noticed in “1201K-1”(48.4%) with an average of 29 seeds per pollinated bunch, followed by “917K-2” (48.2%) with 39.2 seeds per pollinated bunch, “660K-1” (43.5%) with 14.8 seeds per pollinated bunch, “222K-1” (40.9%) with 16.6 seeds per pollinated bunch and “1438K-1” (37.8%) with 19.3 seeds per pollinated bunch (Table 2). These tetraploid hybrids, which are all derived from the Nfuuka clone set, were the most female fertile ones.

There was variation in pollination success when diploid males were crossed with females of different ploidy levels. Highest pollination success for diploid male parents was obtained when crossed with tetraploid females, followed by diploid females and lastly by triploid females (Supplementary Table 1). The highest pollination success was noted in *M. acuminata* subsp. *malaccensis* accession 250 (66.8%) followed by the cultivar “Rose” (66.6%), which is also of *malaccensis* origin. This finding reveals that these two diploids perform better than “Calcutta 4,” which so far has been the most used male parent when screening EAHB for female fertility.

### Seed Germination

The highest percentage of germination was exhibited by seeds from 2*x* × 4*x* crosses (36.0%) followed by seeds from 2*x* × 2*x* crosses (22.8%) (Table 4). Germination success from 3*x* × 2*x* crosses was 11.1%, which is about the same as reported by Vuylsteke et al. (1993) and Talengera et al. (1996), and higher than that of seeds from 4*x* × 2*x* crosses (7.4%). A total of 7887 crosses 4*x* × 2*x* (with 4*x* derived from the EAHB) were made during the period of this study and a total of 217,599 seeds from these crosses were produced.

**Table 4:** Performance of banana crosses as determined by number of seeds, embryos extracted, and embryos germinated (%) from 2005 to 2015 at IITA-Sendusu, Uganda.

Type of cross	Crosses (#)	Seeds ±*SE*	Embryos extracted ±*SE*	Embryos germinated ±*SE*	Germination success (%)
2*x* × 2*x*	114	193.0 ± 32.5	114.8 ± 20	26.2 ± 6.2	22.8
2*x* × 4*x*	102	25.5 ± 5.9	11.1 ± 3.3	4.0 ± 2.3	36.0
3*x* × 2*x*	1225	6.8 ± 1.0	4.5 ± 0.7	0.5 ± 0.1	11.1
3*x* × 4*x*	12	2.3 ± 0.8	1.3 ± 0.5	0.1 ± 0.1	6.0
4*x* × 2*x*	2593	61.0 ± 2.3	43.2 ± 1.7	3.2 ± 0.2	7.4
4*x* × 3*x*	136	10.6 ± 2.1	6.6 ± 1.3	0.8 ± 0.3	12.1
4*x* × 4*x*	261	10.4 ± 2.9	7.2 ± 2.8	0.7 ± 0.1	9.7
All crosses	4443	43.9 ± 1.7	30.3 ± 1.2	2.9 ± 0.2	9.6

### Hybrid Generation, Evaluation and Selection

A group of 27 hybrids (mostly secondary triploids) referred to as NARITAs (Supplementary Table 3) were developed and selected jointly by IITA and NARO (Tushemereirwe et al., 2015). These were sent for evaluation in different agro-ecological zones in East Africa. Nine of the NARITA cultivars had “917K-2” as female parent, and six had “1201K-1” as female parent, while “9128-3” was the male parent in eight of NARITAs, and “SH3217” was a male parent for five NARITAs (Table 5). Out of the 27 NARITA cultivars, NARITA 7 was the first banana cultivar bred from the East African highland banana breeding program to be released in Uganda. Its release name is “KABANA 6H” and evaluation code name “M9,” also nicknamed “Kiwangaazi” by farmers, which means “long-lasting” due to its long plantation life (Nowakunda et al., 2015). It was bred by crossing “1201K-1” (female parent) with “SH3217” (male parent). The female parent (1201K-1) was generated from a cross between cultivar “Nakawere” (female parent) and “Calcutta 4” (male parent). “SH3217” was generated by crossing “SH2095” with “SH2766”. “SH2095” was produced from the cross - [(“Sinwobogi” × “Tjau lagada”) × (“wild *malaccensis*” × “Guyod”)], while “SH2766” was generated from the cross - [“Tjau lagada” × (“wild *malaccensis*” × “Guyod”)] (Supplementary Table 3). Farmers in Uganda have adopted NARITA 7 and it is being grown in farmers’ fields because of its high yield and longevity of plant mats. Three of the NARITA cultivars (NARITA 24, NARITA 25, and NARITA 26) have unknown male and female parents because by the time of their selection from early evaluation trial (EET), their mats had drifted from their original positions, making it difficult to get their true identities from the field map. Molecular tools could be employed in the future to trace their true pedigrees. All the EAHB that generated the tetraploids which were used in the 4*x* × 2*x* crosses producing NARITA cultivars, were from the Nfuuka clone set (Supplementary Table 3).

**Table 5:** Type of cross and number of NARITA hybrid banana cultivars bred.

Female parent	Male parent	NARITAs (#)	NARITA names
917K-2	SH 3217	4	NARITA 5, NARITA 8, NARITA 9, NARITA 10
917K-2	9128-3	2	NARITA 1, NARITA 22
917K-2	SH 3362	2	NARITA 3, NARITA 16
917K-2	7197-2	1	NARITA 14
1201K-1	9128-3	2	NARITA 11, NARITA 12
1201K-1	7197-2	1	NARITA 21
1201K-1	8075-7	1	NARITA 19
1201K-1	SH 3217	1	NARITA 7
1201K-1	SH 3362	1	NARITA 13
660K-1	9128-3	2	NARITA 4, NARITA 15
222K-1	9128-3	1	NARITA 6
222K-1	SH 3362	1	NARITA 27
365K-1	660K-1	1	NARITA 18
401K-1	9128-3	1	NARITA 2
1438K-1	9719-7	1	NARITA 17
Entukura	365K-1	1	NARITA 20
Kazirakwe	7197-2	1	NARITA 23

## Discussion

Pollination success was similar for each month at a 95% confidence level (*P* = 0.501). This implies that pollination of EAHB can be done continuously throughout the year to maximize chances of success. This contrasts with results from Ortiz and Vuylsteke (1995), who demonstrated a seasonal effect in seed set in triploid plantains in Onne – a lowland degraded humid rainforest in southeastern Nigeria, where the highest seed set was noticed at the rain peak in September. They further reported that seeds with tetraploid embryos were most likely obtained after pollinations performed between January and mid-March, which is the end of the dry season. Based on their findings, they concluded that the production of tetraploids is enhanced by pollination under high temperature, high solar radiation, and low relative humidity. These conflicting results may be due to the differences in the climatic conditions between Onne and Sendusu/Namulonge. Onne is at about 5 m.a.s.l. in the coastal humid forest zone of West Africa (4°51′ N, 7°03′ E). Average annual rainfall is 2400 mm and distributed over 10 months (March to December). The temperature is high: averaging 27°C during the warmest months (February, March and April) and 25°C in the coolest month (July) (Ortiz and Vuylsteke, 1995). Sendusu, on the other hand, is located at 1167 m.a.s.l. (00°31′ 47″ N and 32°36′ 9″ E) and with an average temperature of 22°C and average annual rainfall of 1264 mm (Nsubuga et al., 2011). Another possible cause of the difference may be genetic factors as different cultivar groups were used in different locations. In Onne, the study was conducted on plantains (AAB), Yangambi Km5 (AAA) and cooking bananas (ABB), while in Uganda the study was conducted on EAHB and its derived genotypes.

Seed set varied very much among cultivars after crossing EAHB with wild or improved diploids, ranging from 0 to 305 seeds per pollinated bunch. Similar results were observed in the plantain cultivar “Bobby Tannap” crossed with “Calcutta 4,” with number of seeds per bunch ranging from 0 to 219 (Vuylsteke et al., 1993). Ssebuliba et al. (2005), studying female fertility in different EAHB cultivars, observed that cultivars in the Nakabululu clone set had the highest pollination success (49.2%), followed by the Nfuuka clone set (45.1%), supporting our study. They reported, however, that the Mbidde clone set came third with 8.6% pollination success, then the Musakala clone set (3.9%) and lastly the Nakitembe clone set (1.3%). In our study, the Nakitembe clone set was in the third place, followed by the Musakala clone set and lastly the Mbidde clone set. Based on the findings from our study, we would recommend banana breeders to select female parents with good breeding attributes from the Nakabululu and Nfuuka clone sets in order to increase chances of pollination success. In our compilation of data, the seed set of EAHB ranged between 0 to 305 seeds per bunch, with the cultivar “Nakasabira” having the highest seed set per bunch (Table 1). This result differs from earlier research by Ssebuliba et al. (2005), who reported that the seed set for EAHB ranged between 0 to 25 seeds per bunch. Despite the fact that the cultivars “Nakabululu” and “Nakawere” had the highest pollination success, the current crossing scheme at IITA-Sendusu for breeding EAHB does not include these cultivars. Hence, inclusion of these cultivars into the triploid banana crossing scheme needs to be re-considered.

Kitavi et al. (2016) reported that the EAHB represent a set of clonal variants that are all apparently derived from a single original seedling. However, we observed variation among the EAHB in terms of seed set with cultivars in the Mbidde and Musakala clone sets being highly sterile compared to cultivars in other clone sets (Table 1). This warrants further investigation.

Initially, it was thought that the triploidy nature *per se* is the potential cause of sterility in plantain and bananas. However, availability of seed fertile triploid banana and plantains, as reported by Vuylsteke et al. (1993), demonstrated that triploidy does not have to be the cause of sterility in triploid cultivars. Agarwal (1987) suggested that genetic causes, rather than chromosomal irregularities, were responsible for sterility in bananas. Likewise, sterility of female flowers was associated with fruit parthenocarpy, and high auxin concentration in the developing ovary was the common cause of sterility (Dodds, 1945; Dodds and Simmonds, 1948). Furthermore, chromosome aberrations such as translocations and inversions were also reported to cause female and male sterility in bananas (Dodds, 1945). The phenomena causing sterility in *Musa* have been summarized as genomic, chromosomic (numerical and structural) and gene-related, resulting in meiotic, organo-genetic and physiological errors (Dodds, 1945; Dodds and Simmonds, 1948; Agarwal, 1987).

Ssebuliba et al. (2005), while studying fertility of EAHB, found out that abnormalities in pistil morphological traits were possible causes of female sterility. They pointed out style length to be associated with female sterility whereby cultivars with long styles had lower chances of setting seed compared to their counterparts with shorter styles. The possible explanation for this phenomenon is that pollen tubes that travel short distances might have better chances of pollinating ovules than those that travel long distances. They stated, however, that style length alone could not account for female sterility in EAHB, and proposed that other factors related to stigma receptivity, ovule receptivity, fertilization process, and embryo survival after fertilization could be investigated to find out if they play a role in seed set determination.

The tetraploids with the highest number of seeds per bunch and pollination success, namely “917K-2,” “1201K-1,” “660K-1,” “222K-1,” and “1438K-1,” are all derived from the female parents belonging to the Nfuuka clone set. This indicates that cultivars from the Nfuuka clone set produce more hybrid offspring, which allows the breeder to increase both population size and intensity of selection.

The diploids *M. acuminata* subsp. *malaccensis* accession 250 and cultivar “Rose,” are suitable for screening female fertility as they outperform the routinely used “Calcutta 4” in terms of pollination success. However, pollination success *per se* should not be the sole criterion for selecting a good male parent. The genotype of the parents and the combination of characters sought in the progeny need to prevail when selecting the male parent. As *M. acuminata* subsp. *malaccensis* accession 250 and cultivar “Rose” result in hybrids with sub-horizontal bunches, these two accessions are useful when screening for female fertility but they have a rather low breeding value. Nyine et al. (2018) showed that genomic prediction of breeding values may aid in selection of suitable parents and hybrid offspring.

As not all seeds had embryos, the percentage of germination was calculated by the percentage of embryos germinated from the total amount of embryos extracted rather than the total number of seeds received. Ssebuliba et al. (2006) also observed that not all the seeds that appeared phenotypically good had embryos. They found that 72% of the seeds appeared normal phenotypically, but only 59% contained embryos and only 9% of the seeds germinated. This is close to the observed general percentage germination of all crosses combined (9.6%) in our study.

In our study, we observed that seeds from 3*x* × 2*x* crosses had a higher percentage of germination (11.1%) than seeds from 4*x* × 2*x* crosses (7.4%), yet in field evaluation trials there are more hybrids from 4*x* × 2*x* crosses than 3*x* × 2*x*. This is because the 3*x* × 2*x* crosses produce fewer seeds than 4*x* × 2*x* crosses, such that even with the higher percentage germination of the 3*x* × 2*x* seeds, there are still fewer hybrids compared to the 4*x* × 2*x* derived hybrids. Ortiz and Crouch (1997) investigated the efficiency of natural and artificial pollinators in plantain-derived tetraploid hybrids crossed with diploid *Musa* accessions and found out that the germination rate of seeds from natural open pollinations was higher than that of seeds generated from artificial pollinations. There is therefore a need to optimize both the pollination conditions and the protocol for embryo culture to boost the germination level of seeds. It should be noted that the *3x* cultivars in the 4*x* × 3*x* crosses and 3*x* × 4*x* crosses (Table 4) were not EAHB landraces. They were either triploids derived from hybridization of the EAHB, primary tetraploids with wild and improved diploids or triploids imported from other banana groups. Hence, we cannot compare these crosses with 4*x* × 2*x* and 3*x* × 2*x* crosses.

Germinating embryos *in vitro* were grown up, weaned and planted in EET. Twenty EETs were planted during the first two decades, each having an average of 600 genotypes. The most promising hybrids from each EET were selected for further testing in preliminary yield trials (PYT), for growth, bunch, and fruit characteristics during at least two crop cycles (mother plant and first ratoon). The sensory attributes of these selected hybrids were also analyzed. The outstanding hybrids were advanced to multisite trials where their performance was assessed in different agro-ecological zones within Uganda in partnership with NARO. Thereafter the highest performing hybrids were advanced by NARO to on-farm evaluation where they were planted in farmers’ fields and cultivated under farmers’ management. This is the final stage of East African highland banana hybrid evaluation before their release as new cultivars.

The selection of NARITA cultivars was based on having bigger bunches than their parents or grandparents, host plant resistance or tolerance to black sigatoka, and good culinary attributes. Hence, all NARITA cultivars show bunches bigger than their parents and grandparents and are less affected by black sigatoka. These were therefore regarded as the main output of the banana breeding program. From this study, it was revealed that “917K-2” was the female parent that generated the highest number of NARITAs (9) irrespective of the males, while “9128-3” was the male parent that generated the highest number of NARITAs (8) irrespective of the females. However, the female-male combination that gave the highest number of NARITAs was “917K-2 × SH3217,” which generated 4 NARITA cultivars namely; NARITA 5, NARITA 8, NARITA 9, and NARITA 10 (Table 5).

## Conclusion and Recommendations

The success of a *Musa* crossbreeding program depends highly on the production of viable seeds through sexual hybridization (Ortiz and Swennen, 2014). We investigated pollination success over a period of 21 years and found that there was no preference for a particular month in the year, hence pollinations in the EAHB breeding program can be performed all year-round whenever there are flowers available. The diploids *M. acuminata* subsp. *malaccensis* accession 250 and cultivar “Rose” appear to be the most ideal male parents to screen for female fertility.

Considering the low female fertility rates of EAHB (mean number of seeds per bunch between 0 and 1.5) and their derived tetraploids (mean number of seeds per bunch between 0.2 and 39.2) and the occasional high seed set (305 seeds for Nakasabira and 2279 seeds for 917K-2), research is needed on factors influencing fertility, so that pollination conditions can be improved to boost seed set. Likewise, the big discrepancy between seeds harvested and embryos germinated (on average 44 seeds of which 3 embryos germinate), necessitates more research on factors influencing fertilization, seed development and seed germination. This is of particular value for key crosses in EAHB breeding like 3*x* × 2*x* crosses and 4*x* × 2*x* crosses, which yield the primary tetraploid and secondary triploid hybrids, respectively. The seed fertile EAHB cultivars “Nakabululu” and “Nakawere,” with the highest pollination success, are not currently in use and hence may need to be considered by the EAHB breeding program.

Selecting 27 NARITAs reaching an advanced stage of evaluation in the East African region and having only one of them being officially released to farmers in Uganda during the first 21 years of breeding, clearly demonstrates the challenges when breeding bananas, starting from near sterile cultivars. Overcoming sterility barriers and investigating other breeding approaches are required to improve the efficiency of banana breeding programs.

## Author Contributions

MB, RS, BU, AB, HH, MG, and RO contributed to the conception and design of the study. MB, BU, and VA organized the database. MB and RT performed the statistical analysis. MB wrote the draft manuscript, while all co-authors contributed to manuscript revision, read and approved the submitted version.

## Conflict of Interest Statement

The authors declare that the research was conducted in the absence of any commercial or financial relationships that could be construed as a potential conflict of interest.
