# Supplementary material for: Crossbreeding East African Highland Bananas: Lessons Learnt Relevant to the Botany of the Crop After 21 Years of Genetic Enhancement
Source: Front Plant Sci. 2019 Feb 5;10:81. doi: 10.3389/fpls.2019.00081 (PMC6370977; doi:10.3389/fpls.2019.00081)
Supplement: Supplementary file 1 [file Table_1.docx]

**Supplementary Table 1** Pollination success (%) in 20 diploid bred banana hybrids, 5 landraces and 4 wild accessions crossed with 2*x,* 3*x* and 4*x* females from April 1995 to December 2015 at IITA-Sendusu, Uganda

| Male Parent (2*x*) | Crossing with | Crosses (#) | Seeds (#) | Crosses without seed (#) | Highest seed per cross (#) | Seed per cross ± SE | Pollination success (%) |
| --- | --- | --- | --- | --- | --- | --- | --- |
| 861S-1 | 4*x* | 161 | 820 | 100 | 84 | 5.1 ± 0.9 | 37.9 |
| 1297-3 | 2*x* | 70 | 1622 | 46 | 612 | 23.2 ± 9.9 | 34.3 |
|  | 3*x* | 5 | 0 | 5 | 0 | 0 | 0 |
|  | Total | 75 | 1622 | 51 | 612 | 21.6 ± 9.2 | 32.0 |
| 1518-4 | 2*x* | 9 | 14 | 6 | 8 | 1.6 ± 0.9 | 33.3 |
|  | 3*x* | 13 | 0 | 13 | 0 | 0 | 0 |
|  | Total | 22 | 14 | 19 | 8 | 0.6 ± 0.4 | 13.6 |
| 1537K-1 | 4*x* | 251 | 1162 | 150 | 116 | 4.6 ± 0.7 | 40.2 |
| 5105-1 | 2*x* | 98 | 4539 | 66 | 1172 | 46.3 ± 18.2 | 32.7 |
|  | 3*x* | 1757 | 625 | 1611 | 33 | 0.4 ± 0.04 | 8.3 |
|  | 4*x* | 171 | 3467 | 93 | 276 | 20.3 ± 3.4 | 45.6 |
|  | Total | 2026 | 8631 | 1770 | 1172 | 4.3 ± 3.4 | 12.6 |
| 5265-1 | 2*x* | 45 | 220 | 35 | 80 | 4.9 ± 2.3 | 22.2 |
|  | 3*x* | 65 | 0 | 65 | 0 | 0 | 0 |
|  | 4*x* | 277 | 3570 | 131 | 293 | 12.9 ± 2.1 | 47.3 |
|  | Total | 387 | 3790 | 231 | 293 | 9.8 ± 1.6 | 40.3 |
| 5610S-1 | 2*x* | 43 | 660 | 35 | 342 | 15.3 ± 8.8 | 18.6 |
|  | 3*x* | 201 | 107 | 175 | 34 | 0.5 ± 0.2 | 12.9 |
|  | 4*x* | 1008 | 14375 | 516 | 784 | 14.3 ± 6.4 | 48.8 |
|  | Total | 1252 | 15142 | 726 | 784 | 12.1 ± 1.0 | 42.0 |
| 6142-1 | 2*x* | 39 | 1666 | 30 | 1250 | 42.7 ± 32.8 | 23.0 |
|  | 3*x* | 55 | 0 | 55 | 0 | 0 | 0 |
|  | 4*x* | 57 | 35 | 43 | 7 | 0.6 ± 0.2 | 24.6 |
|  | Total | 151 | 1701 | 128 | 1250 | 11.3 ± 8.5 | 15.2 |
| 7197-2 | 2*x* | 99 | 5234 | 67 | 1639 | 52.9 ± 19.0 | 32.3 |
|  | 3*x* | 4559 | 2655 | 3971 | 305 | 0.6 ± 0.1 | 12.9 |
|  | 4*x* | 394 | 21977 | 203 | 1901 | 55.8 ± 7.4 | 48.5 |
|  | Total | 5052 | 29866 | 4241 | 1901 | 5.9 ± 0.7 | 16.1 |
| 8075-7 | 2*x* | 288 | 26110 | 184 | 8000 | 90.7 ± 29.8 | 36.1 |
|  | 3*x* | 5421 | 2474 | 4860 | 104 | 0.5 ± 0.04 | 10.3 |
|  | 4*x* | 532 | 14432 | 322 | 935 | 27.1 ± 3.4 | 39.5 |
|  | Total | 6241 | 43016 | 5366 | 8000 | 6.9 ± 1.4 | 14.0 |
| 8532-1 | 2*x* | 78 | 11462 | 39 | 2492 | 147.0 ± 48.7 | 50.0 |
|  | 3*x* | 96 | 5 | 94 | 3 | 0.05 ± 0.04 | 2.1 |
|  | Total | 174 | 11467 | 133 | 2492 | 66.0 ± 22.4 | 23.6 |
| 8848-1 | 2*x* | 43 | 3059 | 29 | 1354 | 72.8 ± 38.7 | 31.0 |
| 9128-3 | 2*x* | 241 | 4002 | 192 | 1298 | 16.6 ± 6.5 | 20.3 |
|  | 3*x* | 343 | 141 | 326 | 85 | 0.4 ± 0.3 | 5.0 |
|  | 4*x* | 1251 | 16572 | 753 | 405 | 13.2 ± 1.1 | 39.8 |
|  | Total | 1835 | 20715 | 1271 | 1298 | 11.3 ± 1.1 | 30.7 |
| 9719-7 | 2*x* | 152 | 8674 | 102 | 1740 | 57.1 ± 15.2 | 32.9 |
|  | 3*x* | 213 | 19 | 207 | 12 | 0.1 ± 0.1 | 2.8 |
|  | 4*x* | 100 | 2332 | 62 | 408 | 23.3 ± 6.3 | 38.0 |
|  | Total | 465 | 11025 | 371 | 1740 | 23.7 ± 5.3 | 20.2 |
| 9839-1 | 2*x* | 92 | 19750 | 40 | 3499 | 214.7 ± 57.2 | 56.5 |
|  | 3*x* | 29 | 13 | 27 | 9 | 0.4 ± 0.3 | 6.9 |
|  | Total | 121 | 19763 | 67 | 3499 | 163.3 ± 44.2 | 44.6 |
| 02145/1320 | 3x | 35 | 0 | 35 | 0 | 0 | 0 |
|  | 4x | 196 | 750 | 138 | 147 | 3.8 ± 1.0 | 29.6 |
|  | Total | 231 | 750 | 173 | 147 | 3.2 ± 0.9 | 25.1 |
| 10969S-1 | 2*x* | 3 | 0 | 3 | 0 | 0 | 0 |
|  | 3*x* | 5 | 0 | 5 | 0 | 0 | 0 |
|  | 4*x* | 218 | 209 | 199 | 94 | 1.0 ± 0.5 | 8.7 |
|  | Total | 226 | 209 | 207 | 94 | 0.9 ± 0.5 | 8.4 |
| ‘Calcutta 4’ | 2*x* | 362 | 35970 | 219 | 8413 | 99.4 ± 30.6 | 39.5 |
|  | 3*x* | 3107 | 6057 | 2696 | 1345 | 1.9 ± 0.6 | 13.2 |
|  | 4*x* | 98 | 960 | 80 | 601 | 9.8 ± 6.4 | 18.4 |
|  | Total | 3567 | 42987 | 2995 | 8413 | 12.1 ± 3.2 | 16.0 |
| Cv. ‘Rose’  (‘Pisang rejang’) | 2*x* | 37 | 14612 | 24 | 6742 | 394.9 ± 245.0 | 35.1 |
|  | 3*x* | 1046 | 1606 | 869 | 280 | 1.5 ± 0.4 | 16.9 |
|  | 4*x* | 374 | 33340 | 125 | 1150 | 89.1 ± 14.0 | 66.6 |
|  | Total | 1457 | 49558 | 1018 | 6742 | 34.0 ± 6.7 | 30.1 |
| ‘Kokopo’ | 2*x* | 19 | 2 | 18 | 2 | 0.1 ± 0.1 | 5.3 |
|  | 3*x* | 5 | 1 | 4 | 1 | 0.2 ± 0.2 | 20.0 |
|  | 4*x* | 526 | 7091 | 286 | 516 | 13.5 ± 1.9 | 45.6 |
|  | Total | 550 | 7094 | 308 | 516 | 12.9 ± 1.8 | 44.0 |
| ‘Long Tavoy’ | 2*x* | 20 | 2693 | 17 | 1492 | 134.7 ± 88.0 | 15.0 |
|  | 3*x* | 6 | 2 | 5 | 2 | 0.3 ± 0.3 | 16.7 |
|  | 4*x* | 25 | 1131 | 10 | 268 | 45.2 ±13.6 | 60.0 |
|  | Total | 51 | 3826 | 32 | 1492 | 75.0 ± 35.2 | 37.3 |
| *M. acuminata* subsp. *malaccensis* 250 | 2*x* | 12 | 3832 | 8 | 3485 | 319.3 ± 288.7 | 33.3 |
|  | 3*x* | 1089 | 1925 | 807 | 102 | 1.8 ± 0.2 | 25.9 |
|  | 4*x* | 263 | 38439 | 88 | 2279 | 146.2 ± 15.0 | 66.8 |
|  | Total | 1364 | 44196 | 903 | 3485 | 32.4 ± 4.1 | 33.7 |
| *Musa balbisiana* | 2*x* | 10 | 488 | 6 | 245 | 48.8 ± 29.3 | 40.0 |
|  | 3*x* | 20 | 1851 | 14 | 1823 | 92.6 ± 91.1 | 30.0 |
|  | 4*x* | 14 | 343 | 6 | 254 | 24.5 ± 17.8 | 57.1 |
|  | Total | 44 | 2682 | 26 | 1823 | 61.0 ± 41.9 | 40.9 |
| ‘Pisang lilin’ | 2*x* | 244 | 4195 | 179 | 1410 | 17.2 ± 6.4 | 26.6 |
|  | 3*x* | 390 | 10 | 383 | 2 | 0.02 ± 0.01 | 1.8 |
|  | 4*x* | 54 | 263 | 41 | 52 | 4.9 ± 2.3 | 24.1 |
|  | Total | 688 | 4468 | 603 | 1410 | 6.5 ± 2.3 | 12.3 |
| SH 3142 | 2*x* | 81 | 155 | 73 | 116 | 1.9 ± 1.5 | 9.9 |
|  | 3*x* | 521 | 163 | 503 | 58 | 0.3 ± 0.1 | 3.5 |
|  | 4*x* | 621 | 5662 | 379 | 217 | 9.1 ± 0.9 | 39.0 |
|  | Total | 1223 | 5980 | 955 | 217 | 4.9 ± 0.5 | 21.9 |
| SH 3217 | 2*x* | 93 | 704 | 79 | 427 | 7.6 ± 4.7 | 15.1 |
|  | 3*x* | 554 | 597 | 509 | 398 | 1.1 ± 0.7 | 8.1 |
|  | 4*x* | 888 | 33463 | 429 | 590 | 37.7 ± 2.6 | 51.7 |
|  | Total | 1535 | 34764 | 1017 | 590 | 22.6 ± 1.6 | 33.7 |
| SH 3362 | 2*x* | 21 | 16 | 18 | 8 | 0.8 ± 0.5 | 14.3 |
|  | 3*x* | 486 | 106 | 460 | 15 | 0.3 ± 0.1 | 5.3 |
|  | 4*x* | 555 | 20990 | 288 | 678 | 37.9 ± 3.4 | 48.1 |
|  | Total | 1062 | 21112 | 766 | 678 | 19.9 ± 1.9 | 27.9 |
| ‘Tuu Gia’ | 4*x* | 94 | 534 | 68 | 136 | 5.7 ± 2.0 | 27.7 |
| ‘Yalim’ | 2*x* | 13 | 0 | 13 | 0 | 0 | 0 |
|  | 3*x* | 6 | 1 | 5 | 1 | 0.2 ± 0.2 | 16.7 |
|  | 4*x* | 138 | 186 | 112 | 39 | 1.3 ± 0.5 | 18.8 |
|  | Total | 157 | 187 | 130 | 39 | 1.2 ± 0.4 | 17.2 |
