# Supplementary material for: Crossbreeding East African Highland Bananas: Lessons Learnt Relevant to the Botany of the Crop After 21 Years of Genetic Enhancement
Source: Front Plant Sci. 2019 Feb 5;10:81. doi: 10.3389/fpls.2019.00081 (PMC6370977; doi:10.3389/fpls.2019.00081)
Supplement: Supplementary file 2 [file Table_2.docx]

**Supplementary Table 2** Pedigrees and breeding attributes for diploid males

| Diploid | Female parent | Male parent | Target breeding traits |
| --- | --- | --- | --- |
| 861S-1 | ‘Namwezi’ | ‘Calcutta 4’ | ‘Matooke' qualities, Black sigatoka tolerance |
| 1297-3 | ‘French reversion’ | ‘Calcutta 4’ | Black sigatoka resistance |
| 1518-4 | ‘Bobby Tannap’ | ‘Calcutta 4’ | Black sigatoka resistance |
| 1537K-1 | ‘Kabucuragye’ | ‘Calcutta 4’ | Black sigatoka resistance |
| 5105-1 | ‘Pisang lilin’ | ‘Calcutta 4’ | Black sigatoka resistance |
| 5265-1 | ‘Tjau Lagada’ | ‘Calcutta 4’ | Black sigatoka resistance |
| 5610S-1 | ‘Kabucuragye’ | 7197-2 | 'Matooke' qualities, Black sigatoka tolerance |
| 6142-1 | ‘Nyamwihogora’ | ‘Long Tavoy’ | 'Matooke' qualities, Black sigatoka tolerance |
| 7197-2 | SH 3362 | ‘Long Tavoy’ | Black sigatoka resistance, nematode resistance, |
| 8075-7 | SH 3362 | ‘Calcutta 4’ | Black sigatoka resistance, nematode resistance |
| 8532-1 | ‘Heva’ | ‘Calcutta 4’ | Black sigatoka resistance |
| 8848-1 | ‘Calcutta 4’ | ‘Uwati’ | Black sigatoka resistance |
| 9128-3 | ‘Tjau lagada’ | ‘Pisang lilin’ | Big bunch, *Fusarium* wilt resistance |
| 9719-7 | ‘Madang’ | ‘Calcutta 4’ | Black sigatoka resistance |
| 9839-1 | ‘Calcutta 4’ | ‘Padri’ | Black sigatoka resistance |
| 02145/1320 | Open pollinated ‘Zebrina GF’ |  | Dwarfism, Black sigatoka resistance |
| 10969S-1 | 376K-7 | 5105-1 | 'Matooke' qualities, Black sigatoka tolerance |
| ‘Calcutta 4’ |  |  | Black sigatoka resistance, weevil resistance, nematodes tolerance |
| Cv. ‘Rose’ (‘Pisang rejang’) |  |  | Resistance to *Fusarium* wilt, tolerance to Black sigatoka, parthenocarpic fruits. |
| ‘Kokopo’ |  |  | Parthenocarpic fruits, yellow pulp, quick maturity |
| ‘Long Tavoy’ |  |  | Black sigatoka resistance, weevil resistance, nematodes tolerance |
| M. *acuminata* subsp. *malaccensis* 250 |  |  | Black sigatoka resistance, weevil resistance, nematodes tolerance |
| *Musa balbisiana* |  |  | Drought tolerance, Black sigatoka resistance, weevil resistance, nematodes tolerance |
| ‘Pisang lilin’ |  |  | Parthenocarpic fruits, tolerance to Fusarium wilt, tolerance to black sigatoka |
| SH 3142 | Intermating ‘Pisang Jari Buaya’ |  | Nematode resistance, big bunch |
| SH 3217 | SH 2095 [(‘Sinwobogi’ X ‘Tjau lagada’) X (wild *malaccensis* X ‘Guyod’)] | SH 2766 [‘Tjau lagada’ X (wild *malaccensis* X ‘Guyod’)] | Big bunch |
| SH 3362 | SH 3217 | SH 3142 | Nematode resistance, big bunch |
| ‘Tuu Gia’ |  |  | Parthenocarpic fruit, Black sigatoka tolerance |
| ‘Yalim’ |  |  | Parthenocarpic fruit, yellow pulp |
