# Supplementary material for: Crossbreeding East African Highland Bananas: Lessons Learnt Relevant to the Botany of the Crop After 21 Years of Genetic Enhancement
Source: Front Plant Sci. 2019 Feb 5;10:81. doi: 10.3389/fpls.2019.00081 (PMC6370977; doi:10.3389/fpls.2019.00081)
Supplement: Supplementary file 3 [file Table_3.docx]

**Supplementary Table 3** Pedigrees of NARITA cultivars

| **NARITA Cultivar** | **Female parent** | **Male parent** | **Pedigrees for the female parents** | **Pedigrees for the male parents** |
| --- | --- | --- | --- | --- |
| **NARITA 1** | 917k-2 | 9128-3 | (Enzirabahima × Calcutta 4) | (Tjau lagada × Pisang lilin) |
| **NARITA 2** | 401k-1 | 9128-3 | (Entukura × Calcutta 4) | (Tjau lagada × Pisang lilin) |
| **NARITA 3** | 917k-2 | SH 3362 | (Enzirabahima × Calcutta 4) | (SH 3217 × SH 3142), SH 3217(SH 2095 × SH 2766), SH 2095[(Sinwobogi × Tjau lagada) × (wild *malaccensis* × Guyod)], SH 2766 [Tjau lagada × (wild *malaccensis* × Guyod)], SH 3142 (Intermating Pisang Jari Buaya) |
| **NARITA 4** | 660k-1 | 9128-3 | (Enzirabahima × Calcutta 4) | (Tjau lagada × Pisang lilin) |
| **NARITA 5** | 917k-2 | SH 3217 | (Enzirabahima × Calcutta 4) | (SH 2095 × SH 2766), SH 2095[(Sinwobogi × Tjau lagada) × (wild *malaccensis* × Guyod)], SH 2766 [Tjau lagada × (wild *malaccensis* × Guyod)] |
| **NARITA 6** | 222k-1 | 9128-3 | (Nfuuka × Calcutta 4) | (Tjau lagada × Pisang lilin) |
| **NARITA 7** | 1201k-1 | SH 3217 | (Nakawere × Calcutta 4) | (SH 2095 × SH 2766), SH 2095[(Sinwobogi × Tjau lagada) × (wild *malaccensis* × Guyod)], SH 2766 [Tjau lagada × (wild *malaccensis* × Guyod)] |
| **NARITA 8** | 917k-2 | SH 3217 | (Enzirabahima × Calcutta 4) |  |
| **NARITA 9** | 917k-2 | SH 3217 |  |  |
| **NARITA 10** | 917k-2 | SH 3217 |  |  |
| **NARITA 11** | 1201k-1 | 9128-3 | (Nakawere × Calcutta 4) | (Tjau lagada × Pisang lilin) |
| **NARITA 12** | 1201k-1 | 9128-3 |  |  |
| **NARITA 13** | 1201k-1 | SH 3362 | (Nakawere × Calcutta 4) | (SH 3217 × SH 3142), SH 3217(SH 2095 × SH 2766), SH 2095[(Sinwobogi × Tjau lagada) × (wild *malaccensis* × Guyod)], SH 2766 [Tjau lagada × (wild *malaccensis* × Guyod)], SH 3142 (Intermating Pisang Jari Buaya) |
| **NARITA 14** | 917k-2 | 7197-2 | (Enzirabahima × Calcutta 4) | (SH 3362 × Long Tavoy), SH 3362 (SH 3217 × SH 3142), SH 3217(SH 2095 × SH 2766), SH 2095[(Sinwobogi × Tjau lagada) × (wild *malaccensis* × Guyod)], SH 2766 [Tjau lagada × (wild *malaccensis* × Guyod)], SH 3142 (Intermating Pisang Jari Buaya) |
| **NARITA 15** | 660k-1 | 9128-3 |  | (Tjau lagada × Pisang lilin) |
| **NARITA 16** | 917k-2 | SH 3362 | (Enzirabahima × Calcutta 4) | (SH 3217 × SH 3142), SH 3217(SH 2095 × SH 2766), SH 2095[(Sinwobogi × Tjau lagada) × (wild *malaccensis* × Guyod)], SH 2766 [Tjau lagada × (wild *malaccensis* × Guyod)], SH 3142 (Intermating Pisang Jari Buaya) |
| **NARITA 17** | 1438k-1 | 9719-7 | (Entukura × Calcutta 4) | (Madang × Calcutta 4) |
| **NARITA 18** | 365k-1 | 660k-1 | (Kabucuragye × Calcutta 4) | (Enzirabahima × Calcutta 4) |
| **NARITA 19** | 1201k-1 | 8075-7 | (Nakawere × Calcutta 4) | male parent 8075-7 (SH 3362 × Calcutta 4), SH 3362 (SH 3217 × SH 3142), SH 3217(SH 2095 × SH 2766), SH 2095[(Sinwobogi × Tjau lagada) × (wild *malaccensis* × Guyod)], SH 2766 [Tjau lagada × (wild *malaccensis* × Guyod)], SH 3142 (Intermating Pisang Jari Buaya) |
| **NARITA 20** | Entukura | 365k-1 | Entukura | (Kabucuragye × Calcutta 4) |
| **NARITA 21** | 1201k-1 | 7197-2 | (Nakawere × Calcutta 4) | (SH 3362 × Long Tavoy), SH 3362 (SH 3217 × SH 3142), SH 3217(SH 2095 × SH 2766), SH 2095[(Sinwobogi × Tjau lagada) × (wild *malaccensis* × Guyod)], SH 2766 [Tjau lagada × (wild *malaccensis* × Guyod)], SH 3142 (Intermating Pisang Jari Buaya) |
| **NARITA 22** | 917k-2 | 9128-3 | (Enzirabahima × Calcutta 4) | (Tjau lagada × Pisang lilin) |
| **NARITA 23** | Kazirakwe | 7197-2 | Kazirakwe | (SH 3362 × Long Tavoy), SH 3362 (SH 3217 × SH 3142), SH 3217(SH 2095× SH 2766), SH 2095[(Sinwobogi × Tjau lagada) × (wild *malaccensis* × Guyod)], SH 2766 [Tjau lagada × (wild *malaccensis* × Guyod)], SH 3142 (Intermating Pisang Jari Buaya) |
| **NARITA 24** | unknown | unknown | N/A | N/A |
| **NARITA 25** | unknown | unknown | N/A | N/A |
| **NARITA 26** | unknown | unknown | N/A | N/A |
| **NARITA 27** | 222K-1 | SH 3362 | (Nfuuka × Calcutta 4) | (SH 3217 × SH 3142), SH 3217(SH 2095× SH 2766), SH 2095[(Sinwobogi × Tjau lagada) × (wild *malaccensis* × Guyod)], SH 2766 [Tjau lagada × (wild *malaccensis* × Guyod)], SH 3142 (Intermating Pisang Jari Buaya) |
